# The Difference in the Count

**Published:** 1881-04

**Authors:** 


THE DIFFERENCE IN THE COUNT.—We have just read editorials in *The Sanitary Engineer*, and Boston *Medical and Surgical Journal*, on the difference in the United States census computation, and that of the health department of the City of Baltimore; and also the letter of Mr. A. R. Carter, Secretary of our Health Department in the *Sanitary Engineer* in relation thereto, and we think he has clearly the best of the argument If figures do not lie, and the logical inferences and conclusions deducible from them are of any value, then Mr. Carter's premises are certainly secured in regard to his computation of the population of Baltimore. Furthermore, it seems to us, according to Mr. Carter's premises, that a population of 400,000, rather than his estimate of 393,796 inhabitants, would be nearer the correct basis for calculating the percentage of mortality.

Like Mr. Carter, we have the utmost confidence in the integrity of General Walker, Chief of the Census Bureau, and we think, with him also, that had the commissioners done their duty in a proper manner, the population of Baltimore would have *exceeded* his estimate and shown, at least, 400,000 souls. Mr. Carter should be commended rather than censured for resolutely defending the ground which he and his chief have so carefully taken; and we know them both too well to doubt for a moment their integrity and sincerity of action in the premises. Dear as the reputation of Baltimore is to Mr. Carter, we feel assured that he could not be induced to add a figure, nor erase a word concerning her interest or her fame that was not entirely in harmony with his convictions of justice and of right.

